# Utilisation of modern contraceptives by sexually active adolescent girls in Rwanda: a nationwide cross-sectional study

**DOI:** 10.1186/s12905-022-01956-y

**Published:** 2022-09-06

**Authors:** Joseph Kawuki, Ghislaine Gatasi, Quraish Sserwanja, David Mukunya, Milton W. Musaba

**Affiliations:** 1 Centre for Health Behaviours Research, Jockey Club School of Public Health and Primary Care, The Chinese University of Hong Kong, Hong Kong, SAR China; 2 Key Laboratory of Environmental Medicine Engineering, School of Public Health, Southeast University, Nanjing, 210009 Jiangsu Province China; 3 Programmes Department, GOAL, Khartoum, Sudan; 4 Department of Public Health, Busitema University, Mbale, Uganda; 5 Department of Obstetrics and Gynaecology, Busitema University, Mbale, Uganda

**Keywords:** Adolescents, Contraceptive use, Family planning, Rwanda

## Abstract

**Background:**

Modern contraceptive use has been shown to influence population growth, protect women’s health and rights, as well as prevent sexually transmitted infections (STIs) for barrier contraceptive methods such as condoms. The present study aimed at assessing the level of utilization and factors associated with modern contraceptive use among sexually active adolescent girls in Rwanda.

**Methods:**

We used secondary data from the Rwanda Demographic and Health Survey (RDHS) 2020 data of 539 sexually active adolescent girls (aged 15 to 19 years). Multistage stratified sampling was used to select study participants. We conducted multivariable logistic regression to assess the association between various socio-demographics and modern contraceptive use using SPSS version 25. Modern contraception included the use of products or medical procedures that interfere with reproduction from acts of sexual intercourse.

**Results:**

Of the 539 sexually active girls, only 94 (17.4%, 95% CI: 13.8–20.1) were using modern contraceptives. Implants (69.1%) and male condoms (12.8%) were the most used options. Modern contraceptive use was positively associated with older age (AOR = 10.28, 95% CI: 1.34–78.70), higher educational level (AOR = 6.98, 95% CI: 1.08–45.07), history of having a sexually transmitted infection (AOR = 8.27, 95% CI: 2.54–26.99), working status (AOR = 1.72, 95% CI: 1.03–2.88) and being from a female-headed household (AOR = 1.96, 95% CI: 1.12–3.43). However, not being in a union (AOR = 0.18, 95% CI: 0.10–0.35) and region (AOR = 0.28, 95% CI: 0.10–0.80) had negative associations.

**Conclusions:**

To promote utilisation of modern contraceptives, family planning campaigns need to place more emphasis on the younger, unmarried adolescents, as well as those with lower educational levels. Consideration of household and regional dynamics is also highlighted.

## Introduction

The use of modern contraceptives is essential for protecting women’s health and rights, reducing population growth, and promoting women’s empowerment and economic development [1]. Furthermore, barrier contraceptive methods like condoms are crucial for preventing sexually transmitted infections [1, 2]. Low and middle-income countries (LMICs) have the highest unmet need for modern contraception as over 200 million women who do not want to conceive are not using a modern contraceptive method [3, 4]. This might partly explain the annual 111 million unintended pregnancies and 35 million unsafe abortions occurring in LMICs [5]. Access to modern contraception and adequate care for all women could reduce unintended pregnancies by 68%, unplanned births by 71%, unsafe abortions by 72% and maternal mortality by 62% [4, 5].

Sub-Saharan Africa (SSA) has the highest rate of teenage pregnancy and over a third of teenage pregnancies end up as unsafe abortions yet the region also reports the lowest utilization of modern contraceptives [6]. Complications related to pregnancy are the major causes of maternal deaths among adolescents aged 15–19 years in LMICs [6, 7]. Amouzou et al*.* analyzed national data on coverage of maternal and reproductive health indicators in 58 LMICs and showed that adolescents had lower access and utilization of family planning (FP) interventions than women aged 20–49 years; had the slowest rate of improvement in coverage for SRH (between 2000 and 2017) and children born to teenage mothers were significantly disadvantaged in terms of receiving timely illness treatment and immunisation [8].

Although the East African Community (EAC) region in which Rwanda belongs has registered progress in access to SRH, the region still has major gaps in access and quality of SRH services with a very high unmet need for contraception, especially among adolescents [6, 9, 10]. Despite the government of Rwanda's youth-friendly policies and projects such as the *Isange One-Stop Centers* and *Youth-friendly corners* in schools and health facilities that promote SRH to young Rwandans, urban areas such as Kigali are increasingly experiencing unwanted adolescent pregnancies, risky sexual behaviours, and rising HIV infection [9, 11]. The Rwanda demographic health survey (RDHS) 2020 further reports that 15% of adolescents have begun childbearing at the age of 19 years [12]. Moreover, there is limited information on contraceptive use among adolescents in Rwanda, where most of the available studies have looked at women in general [4, 10, 13–17]. In order to formulate evidence-based policies aimed at increasing the utilization of modern contraceptives, there is a need for more recent adolescent-focused studies. Hence our study aimed at assessing the level and associated factors of modern contraceptive use among sexually active adolescent girls in Rwanda using the most recent 2020 RDHS. Our findings might be vital in informing targeted interventions and policies aimed at improving access and utilisation of modern contraceptives.

## Methods

### Study sampling and participants

We used the 2019–20 Rwanda Demographic Survey (RDHS) for this analysis. It employed a two-stage sample design, with the first stage involving cluster selection consisting of enumeration areas (EAs) [12]. The second stage involved systematic sampling of households in all the selected EAs leading to a total of 13,005 households [12]. In particular, the data used in this analysis were from the household and the woman’s questionnaires.

During this survey, the data collection period was from November 2019 to July 2020, taking longer than expected due to the COVID-19 pandemic restrictions [12]. Women aged 15–49 years who were either permanent residents of the selected households or visitors who stayed in the household the night before the survey were eligible to be interviewed. Out of the total 13,005 households that were selected for the survey, 12,951 were occupied and 12,949 were successfully interviewed leading to a 100% response rate [12]. The eligible sample was 14,675 women aged 15–49, but 14,634 women were successfully interviewed leading to a 99.7% response rate [12]. This analysis included only adolescent girls aged 15–19 years interviewed during the survey, and from the selected households (n = 3258). For precision of contraceptive use, we focused on the sexually active adolescent girls, whose sample was 539.

### Variables

#### Dependent variables

The study outcome variable was the usage of modern contraceptive methods, and this was a binary variable directly coded yes or no. Modern contraception was considered as the use of a product or medical procedure that interferes with reproduction from acts of sexual intercourse [18] and included implants/Norplant, male condoms, injections, and pills.

#### Explanatory variables

We included possible determinants of modern contraceptive use based on the available literature and data [19–24]. Thirteen (13) variables were considered and of these, two were community-level factors that included; place of residence (categorized into rural and urban), and region of residence (categorized into Kigali, South, West, East and North). Four household-level factors included; household size which was classified into “less than six” and “six and above”, sex of household head (classified as female and male), wealth index (categorized into five quintiles that ranged from the poorest to the richest quintile), and health insurance (categorised as yes and no). Wealth index was calculated by RDHS from information on household asset ownership using Principal Component Analysis [12]. Seven individual-level factors were also considered in the analysis, including; age (categorized as 15, 16, 17, 18 and 19 years), educational level (grouped as no education, primary, secondary and tertiary), working status (working and not working), being in a union (living with partner and not in a union), religion (Catholic, Protestant, and others), history of having an STI in last 12 months (yes and no), and exposure to news, radio and television (yes and no).

### Statistical analysis

We applied the DHS sample weights to account for the unequal probability sampling in different strata and ensure the representativeness of the study results [25, 26]. We used SPSS (version 25.0) statistical software complex samples package incorporating the following variables in the analysis plan to account for the multistage sample design inherent in the RDHS dataset: individual sample weight, sample strata for sampling errors/design, and cluster number [12, 25]. Frequency distributions were used to describe the background characteristics of the sexually active adolescent girls; where frequencies and proportions/percentages for categorical dependent and independent variables have been presented. Bivariable logistic regression was then conducted to assess the association of each predictor variable with modern contraceptive use, and we presented crude odds ratio (COR), 95% confidence interval (CI) and p-values. Independent variables found significant at a p-value less than 0.25 were then included in the multivariable model. Moreover, independent variables that were non-significant at the bivariable analysis level but were associated with contraceptive use in previous studies were also included in the multivariable logistic regression model. Adjusted odds ratios (AOR), 95% confidence intervals (CI) and p-values were calculated and presented, with a statistical significance level set at p-value < 0.05.

Since a small fraction of the adolescent girls was sexually active (16.5%, 539/3258), we conducted a sensitivity analysis where we considered all adolescent girls regardless of their sexual activity to examine any differences in predictor variables of modern contraceptive use. All socio-demographic variables in the model were assessed for multi-collinearity, which was considered present if the variables had a variance inflation factor (VIF) greater than 10 [27]. However, none of the variables had a VIF above 3.

## Results

A total of 539 sexually active adolescent girls were included in this analysis (Table 1). The majority were aged 18 and 19 years (62.4%), had primary education (63.6%), were working (54.5%), were rural residents (78.4%), and were from male-headed households (67.0%) of less than 6 household members (55.9%). Moreover, the majority of the respondents had health insurance (79.9%), exposure to radio (80.5%) and television (52.3%), but no exposure to newspapers (70.4%), as detailed in Table 1.

**Table 1:** Background characteristics of Sexually active Rwandan adolescents aged 15 to 19 years as per the 2020 Rwanda demographic health survey

Characteristics	Frequency (%), N = 539
*Age (years)*	
15	39 (7.2)
16	59 (10.9)
17	104 (19.3)
18	127 (23.5)
19	210 (38.9)
*Education level*	
Tertiary	4 (0.7)
Secondary	178 (33.0)
Primary	342 (63.6)
No education	15 (2.8)
*Working status*	
Working	294 (54.5)
Not working	245 (45.5)
*Being in a union*	
Living with partner	73 (13.6)
Not in a union	465 (86.4)
*Religion*	
Catholic	204(37.9)
Protestant	242(45.0)
Others	92(17.1)
*Health insurance coverage*	
Yes	431 (79.9)
No	108 (20.1)
*History of STI*	
Yes	14 (2.6)
No	524 (97.4)
*Exposure to newspapers*	
Yes	159 (29.6)
No	379 (70.4)
*Exposure to radio*	
Yes	433 (80.5)
No	105 (19.5)
*Exposure to television*	
Yes	282 (52.3)
No	257 (47.7)
*Wealth index*	
Richest	148 (27.5)
Richer	92 (17.1)
Middle	100 (18.6)
Poorer	99 (18.4)
Poorest	99 (18.4)
*Household size*	
Less than 6	301 (55.9)
6 and above	237 (44.1)
*Sex of household head*	
Male	361 (67.0)
Female	178 (33.0)
*Residence*	
Urban	116 (21.6)
Rural	422 (78.4)
*Region*	
Kigali	83 (15.5)
West	100 (18.6)
East	172 (32.0)
North	68 (12.6)
South	115 (21.3)

Of the 539 sexually active girls, only 94 (17.4%, 95% CI: 13.8–20.1) were using modern contraceptives, and implants (69.1%) and male condoms (12.8%) were the most used options, as shown in Table 2.

**Table 2:** Types of modern contraceptives used by sexually active adolescent girls, as per 2020 RDHS

Modern contraceptive use	Frequency (%), N = 539
Yes	*94 (17.4), (95% CI = 13.8–20.1)*
No	445 (82.6)
Type of contraceptive	Frequency (%), N = 94
Implants/Norplant	65 (69.1)
Male condom	12 (12.8)
Injections	9 (9.6)
Pills	8 (8.5)

*CI* confidence interval,* RDHS*  Rwanda demographic health survey

### Factors associated with modern contraceptive use

Results of the bivariable analysis with their respective crude odds ratios and p-values are detailed in Table 3. In the final multiple logistic regression model, the factors found significantly associated with contraceptive use were; age, educational level, history of having a sexually transmitted infection (STI), being in a union, and region.

**Table 3:** Factors associated with modern contraceptive use among sexually active adolescent girls as per the 2020 RDHS

Variable	Crude odds ratio, COR (95% CI)	*p*-value*	Adjusted odds ratio, AOR (95% CI)	*p*-value**
*Age (years)*		**0.002**		**0.031**
15	1		1	
16	2.97(0.32–27.90)		2.88(0.29–28.31)	
17	6.37(0.78–52.09)		5.76(0.70–47.43)	
18	9.29(1.198–72.069)		7.41(0.93–58.85)	
19	13.50(1.77–103.06)		**10.28(1.34–78.70)**	
*Education level*		0.269		**0.027**
No education	1		1	
Primary	1.26(0.32–5.02)		1.88(0.38–9.37)	
Secondary	0.77(0.20–3.01)		1.54(0.30–8.056)	
Tertiary	1.93(0.22–16.92)		**6.98(1.08–45.07)**	
*Working status*		**0.012**		0.240
Not working	1		1	
Working	1.79(1.14–2.83)		1.40(0.80–2.48)	
*Being in a union*		** < 0.001**		** < 0.001**
Living with partner	1		1	
Not in a union	0.12(0.07–0.22)		**0.18(0.10–0.35)**	
*Religion*		0.421		0456
Catholic	1		1	
Protestant	1.09(0.64–1.86)		1.22(0.67–2.23)	
Others	1.49(0.81–2.76)		1.60(0.76–3.37)	
*Health insurance* *coverage*		0.912		0.926
No	1		1	
Yes	1.03(0.60–1.78)		0.98(0.47–1.97)	
*History of STI*		** < 0.001**		**0.001**
No	1		1	
Yes	7.18(2.41–21.45)		**8.27(2.54–26.10)**	
*Exposure to newspapers*		0.402		0.096
No	1		1	
Yes	1.22(0.77–1.94)		1.68(0.913.09	
*Exposure to radio*		**0.187**		0.084
No	1		1	
Yes	0.690(0.398–1.20)		0.55(0.27–1.08)	
*Exposure to television*		**0.017**		0.483
No	1		1	
Yes	0.56(0.35–0.90)		0.79(0.41–1.53)	
*Wealth index*		**0.001**		0.099
Poorest	1		1	
Poorer	1.01(0.53–1.92)		1.04(0.48–2.26)	
Middle	1.71(0.90–3.24)		1.78(0.75–4.20)	
Richer	0.62(0.31–1.25)		0.82(0.32–2.07)	
Richest	0.31(0.14–0.68)		0.51(0.20–1.32)	
*Household size*		**0.014**		0.746
6 and above	1		1	
Less than 6	1.86(1.13–3.05)		0.90(0.48–1.68)	
*Sex of household head*		0.945		
Male	1		1	0.130
Female	0.98(0.60–1.61)		1.60(0.87–2.93)	
*Residence*		**0.152**		
Rural	1		1	0.248
Urban	0.651(0.36–1.17)		1.54(0.74–3.19)	
*Region*		**0.001**		**0.016**
South	1		1	
North	1.28(0.64–2.56)		1.42(0.58–3.44)	
East	1.25(0.70–2.25)		1.23(0.61–2.48)	
West	0.62(0.26–1.52)		0.57(0.23–1.40	
Kigali	0.20(0.08–0.52)		**0.28(0.10–0.80)**	

Bold = significant, * = significant at 0.25, ** = significant at 0.05, CI = confidence interval, RDHS = Rwanda demographic health survey

Compared to girls of 15 years of age, those of 19 years were 10.28 (95% CI: 1.34–78.71) times more likely to use modern contraceptives, while those with tertiary education were 6.98 (95% CI: 1.08–45.07) times more likely to use contraceptives compared to their counterparts with no education. Adolescent girls with a history of an STI within the last 12 months were also 8.27 (95% CI: 2.54–26.99) times more likely to use modern contraceptives compared to those with no STI history. However, unmarried adolescents (ie not in union) were 82% less likely to use modern contraceptives compared to adolescents living with partners (AOR = 0.18, 95% CI: 0.10–0.35), and those residing in Kigali were 72% less likely to use contraceptives compared to their fellows in the Southern region (AOR = 0.28, 95% CI: 0.099–0.802).

### Sensitivity analysis considering all adolescent girls regardless of sexual activity

Results of sensitivity analysis are detailed in Table 4, with some changes noted in the significant predictor variables. Compared to the first analysis, educational level became non-significant on sensitivity analysis and two more factors, that is work status and sex of household head become significant predictors of modern contraceptive use. The other variables remained significant with minor changes noted only in the magnitude of associations.

**Table 4:** Sensitivity analysis, considering all adolescent girls, n = 3258

Variable	Adjusted odds ratio, AOR (95% CI)	*p*-value**
*Age (years)*		** < 0.001**
15	1	
16	4.71(0.52–42.57)	
17	**12.87(1.63–101.45)**	
18	**22.24(2.88–171.93)**	
19	**34.37(4.50–262.40)**	
*Education level*		0.250
No education	1	
Primary	1.71(0.32–9.09)	
Secondary	0.91(0.17–4.75)	
Tertiary	5.08(0.61–42.32)	
*Working status*		**0.038**
Not working	1	
Working	**1.72(1.03–2.88)**	
*Being in a union*		** < 0.001**
Living with partner	1	
Not in a union	**0.03(0.01–0.06)**	
*Religion*		0.405
Catholic	1	
Protestant	1.11(0.65–1.89)	
Others	1.61(0.80–3.23)	
*Health insurance coverage*		0.395
No	1	
Yes	0.76(0.40–1.44)	
*History of STI*		** < 0.001**
No	1	
Yes	**38.89(10.28–147.10**	
*Exposure to newspapers*		0.242
No	1	
Yes	1.37(0.81–2.34)	
*Exposure to radio*		0.186
No	1	
Yes	0.65(0.34–1.23)	
*Exposure to television*		0.945
No	1	
Yes	0.98(0.55–1.76)	
*Wealth index*		0.179
Poorest	1	
Poorer	1.17(0.51–2.68)	
Middle	2.02(0.87–4.64)	
Richer	1.23(0.52–2.95)	
Richest	0.73(0.26–2.02)	
*Household size*		0.843
6 and above	1	
Less than 6	1.06(0.58–1.94)	
*Sex of household head*		**0.019**
Male	1	
Female	**1.96(1.12–3.43)**	
*Residence*		
Rural	1	0.353
Urban	1.37(0.70–2.70)	
*Region*		**0.011**
South	1	
North	1.02(0.46–2.27)	
East	1.27(0.66–2.46)	
West	0.53(0.23–1.21)	
Kigali	**0.28(0.10–0.77)**	

Bold = significant, ** = significant at 0.05, CI = confidence interval

Working adolescents had 1.72 (95% CI: 1.03–2.88) more odds of using modern contraceptives compared to those not working, and those from female-headed households had 1.96 (95% CI: 1.12–3.43) more odds of using contraceptives compared to their counterparts in male-headed households.

Compared to the first analysis of only sexually active adolescents, sensitivity analysis results clearly indicate that modern contraceptive use increases with age, a significant trend noted in age groups of 17, 18 and 19 years (Table 4).

## Discussion

Contraceptive use among adolescents was very low (17.4%), similar to Mali (17.1%) [28], but higher than Uganda (9.4%) [6], Benin (8.5%) [29], Nigeria (14.8%) [30], Nepal (11.9%) [31] and Zambia (12%) [32], according to previous studies. Nonetheless, the Government of Rwanda has put considerable efforts in family planing and reducing unwanted pregnancies as part of its 2030 Family Planing country commitment agenda. Evidence from RDHS shows that modern contraceptives are freely available to adolescent girls from government health facilities [33–35]. However, reasons for the low usage cited among adolescents include; not being married, infrequent sex and not having sex among the unmarried, and menses not returned after giving birth, breastfeeding and fear of side-effects or health concerns among those in unions [35]. However other factors such as lack of awareness and limited sex education in schools and households, misconceptions regarding contraceptive effects on reproduction, negative cultural implications and poor access to services especially in rural areas should be investigated and addressed [36, 37]. In addition, youth access to quality sexual and reproductive services should be expanded since it is a vital component of the sustainable development goals [38], which have less than 10 years to be achieved.

Even when compared to male condoms (12.8%), implants (69.1%) were by far the most popular modern contraceptive method among sexually active Rwandan female youth. This is surprising given that male condoms are non-invasive and more readily available in small shops and local pharmacies as opposed to implants and other modern methods, which require a visit to a health facility and are frequently preceded by a fear of unwanted effects including fertility interference due to their hormonal nature [39–41]. These findings contradict *Dennis *et al*.* (2017), who argue that there is a shift from condoms and pills, which are short-term methods, to injectable contraception and much lower use of long-term methods such as implants and IUD, the reason being that at such a young age, the majority of women are unmarried and having infrequent sex, and thus opting for methods that are easier to use and stop as needed [42]. Nonetheless, our finding could perhaps be explained by the increased availability of implants over other methods, and the inherent convenience advantage of implants due to their longer protective period, thus no need to use/replace them more frequently.

Age, level of education, being in a union, region of residence and history of having a sexually transmitted infection (STI) were all associated with the use of modern contraceptive methods. The odds of using modern contraception increased with age, a factor that has previously been linked with modern contraceptive use [43, 44]. This is not surprising given that older adolescents have more maturity and risk awareness than younger adolescents and are thus more inclined to avoid the negative ramifications of not using it [43, 44]. Another reason could be that older adolescents have a higher level of education, which has previously been demonstrated to positively impact modern contraceptive use [1, 30, 44, 45]. In this study, young women with primary, secondary, and university education were all more likely to use contraception than those with no education, demonstrating that a lack of education correlates with reduced opportunity to learn about modern contraception. Education has been related to women's empowerment and increased awareness of their bodies, reproductive health, and other health information, as well as a better understanding of the value of controlling their birth history regarding their productivity and prospective career development [46, 47]. Therefore, additional efforts are needed to increase girls' education and enhance their awareness about modern contraceptives, as well as general access and comprehension of health-related services, which helps to debunk widespread misconceptions about contraceptive use and effects.

Being in a union was also identified as significantly associated with the use of modern contraceptives. Unmarried adolescents were less likely to be using modern contraceptives compared to those living with partners, a finding previously found in other studies [28, 44, 48, 49]. This is surprising given Rwanda's cultural context, where pregnancy before marriage is highly stigmatized for the girl who "loses *agaciro* or value" and is perceived as a source of shame for her family [50]. Therefore, even though the Rwandan government provides all women with low-cost or free family planning services through community health insurance or *Mutuelles de santé*, unmarried females are at a higher risk of STIs, unexpected pregnancies, and possibly unsafe abortions due to the emphasis placed on virginity and the resulting fear of being judged or labelled for having premarital sex, which prevents girls from seeking modern contraceptives unless they are married [50].

Adolescent girls with a recent history of STI had a higher likelihood of using modern contraception than those with no STI history. A plausible explanation could be the urge to protect themselves from recurrence of infection. Furthermore, they may have boosted their chances of being taught about the benefits of contraceptive methods when receiving care at a health centre.

While female adolescents in the Northern and Eastern regions were more likely to use modern contraception, those in Kigali, Rwanda's capital and predominantly urban city were less likely to use contraception than their counterparts in the Southern region. This contradicts prior research [51, 52]. which revealed that women in urban areas use contraception more than those in rural areas, possibly due to more exposure to numerous streams of information, proximity to health services, and easier access to a wider range of methods to choose from. This study's findings, however, confirm those of a prior study [1], which revealed that contraceptive use is highest in the Northern region of Rwanda, despite a primarily hilly terrain and difficulties in reaching health facilities [53], most likely due to the region's high presence of non-governmental health organizations that emphasize access to sexual and reproductive health services.

A sensitivity analysis that included all female adolescents regardless of sexual activity found identical results to the previous analysis, except that educational level was no longer a significant predictor of modern contraception use. Two new factors emerged: working status and the gender of the household head. Working adolescents were more likely to use modern contraceptives than those not working, a finding also seen elsewhere in similar studies [1, 54] which could be because women who are employed and able to support themselves financially are more likely to be educated and have greater access to contraception and other information. Furthermore, their career duties and goals may be prompting them to regulate and space births. Finally, female adolescents from female-headed households had higher odds of using contraceptives compared to their counterparts in male-headed households. Despite the fact that female-headed households face a variety of personal, familial, and social challenges, a prior study [55] found that becoming the head of the household is also associated with increased social maturity, which may enable mothers to understand the importance of conversing to their children about social issues and conveying health-related information. The influence of household headship on children's and adolescents' health-seeking behaviour should be further explored.

## Strength and limitations

Since the RDHS data used in this study is nationally representative, the results can be generalized to other Rwandan female populations between the ages of 15 and 19 years. DHS data is also known for generating substantial data, with high response rates, high-quality interviewer training, standardized data collection processes across countries, and consistent content across time, allowing these findings to be compared across populations and over time to assess trends [56]. Nevertheless, because the replies are self-reported and cannot be verified, there is a risk of information bias due to the sensitive nature of sexually related topics. Moreover, the cross-sectional methodology used in this study simply allows for assessing the prevalence and investigating the association between exposures and outcomes without drawing any causal inferences.

## Conclusions

In this study, age, level of education, being in a union, residence region, history of a sexually transmitted infection, working status, as well as the gender of the household's head were all found to be associated with modern contraception use among Rwandan female adolescents. As a result, family planning campaigns should place a greater emphasis on younger, unmarried adolescents, as well as those with a lower educational level. This also highlights the need to promote not only the availability or accessibility of contraceptives but also training to address fallacious cultural beliefs that may impede modern contraceptive use among sexually active adolescents, which has been linked to several negative outcome measures that may limit their prospects. This can be achieved if the government and other stakeholders engage more in outreach and community-based projects aimed at improving youth sexual and reproductive health, as well as training to improve healthcare providers' ability to provide youth-friendly services. Family planning strategies and techniques should be adjusted to meet the particular needs of different communities, age groups, marital and working status of women, diverse geographical areas, and so on, in order to reach the largest number of women possible.

## Data Availability

The data set used is openly available upon permission from the MEASURE DHS website (URL: https://www.dhsprogram.com/data/available-datasets.cfm). However, authors are not authorized to share this data set with the public but anyone interested in the data set can seek it with written permission from the MEASURE DHS website (URL: https://www.dhsprogram.com/data/available-datasets.cfm).

## Abbreviations

EA: Enumeration area
AOR: Adjusted odds ratio
LMICs: Low and middle-income countries
SDGs: Sustainable development goals
CI: Confidence interval
COR: Crude odds ratio
DHS: Demographic Health Survey
RDHS: Rwanda Demographic Health Survey
OR: Odds ratio
VIF: Variance inflation factor
WHO: World Health Organization
SPSS: Statistical package for social science
